# Association of SGLT2 inhibitors with lower incidence of death in type 2 diabetes mellitus and causes of death analysis

**DOI:** 10.1038/s41598-022-13760-7

**Published:** 2022-06-16

**Authors:** Mu-Chi Chung, Hui-Tsung Hsu, Chao-Hsiang Chang, Peir-Haur Hung, Po-Jen Hsiao, Laing-You Wu, Ming-Ju Wu, Jeng-Jer Shieh, Chi-Jung Chung

**Affiliations:** 1 Division of Nephrology, Department of Medicine, Taichung Veterans General Hospital, Taichung, Taiwan; 2 Ph.D. Program in Translational Medicine, National Chung Hsing University, Taichung, Taiwan; 3 Rong Hsing Research Center for Translational Medicine, National Chung Hsing University, Taichung, Taiwan; 4 Department of Biotechnology, Asia University, Taichung, Taiwan; 5 Department of Public Health, College of Public Health, China Medical University, No. 100, Sec. 1, Jingmao Rd., Beitun Dist., Taichung City, 406040 Taiwan; 6 Department of Urology, China Medical University and Hospital, Taichung, Taiwan; 7 Department of Internal Medicine, Ditmanson Medical Foundation Chiayi Christian Hospital, Chiayi, Taiwan; 8 Department of Applied Life Science and Health, Chia-Nan University of Pharmacy and Science, Tainan, Taiwan; 9 Institute of Biomedical Sciences, National Chung Hsing University, Taichung, Taiwan; 10 Department of Education and Research, Taichung Veterans General Hospital, Taichung, Taiwan; 11 Department of Medical Research, China Medical University Hospital, Taichung, Taiwan

**Keywords:** Cardiovascular diseases, Endocrine system and metabolic diseases, Drug discovery, Medical research

## Abstract

Sodium-glucose cotransporter 2 inhibitor (SGLT2i) potentially decrease all-cause and cardiovascular death, however, associations with non-cardiovascular death remain unclear. Therefore, we investigated SGLT2i associations with death and the cause of death. We used the Taiwanese National Health Institutes Research database linked to the National Register of Deaths (NRD). Incident type 2 diabetes mellitus (T2DM) patients and propensity score matched T2DM SGLT2i and Dipeptidyl peptidase 4 inhibitor (DPP4i) users were investigated. The index year was the SGLT2i or DPP4i prescription date from May 2016. Patients were followed-up until death or December 2018. Deaths verified by the NRD and grouped accordingly. Multiple Cox proportional hazards models were used. In total, 261,211 patients were included in the population; 47% of the patients were female and the average age was 62 years. The overall incidence of all-cause death was 8.67/1000 patient-years for SGLT2i and 12.41 for DPP4i users during follow-up. After adjusting for potential risk factors in the propensity score matched population, SGLT2i users were associated with lower risks of all-cause death, cardiovascular death, cancer death, and non-cancer, non-vascular death compared with DPP4i-users. For specific death causes, significantly lower death risks from heart disease, cerebrovascular disease, and accidents were associated with SGLT2i-use. SGLT2i benefits for T2DM patients were not different across subgroups. Compared with DPP4i-use, SGLT2i-use for T2DM was associated with lower disease and death risk.

## Introduction

Diabetes mellitus (DM) complications pose a major global health threat^1^. DM patients have a twofold risk of all-cause death when compared with other non-DM patients^2^. The trend in all-cause death has declined considerably in recent decades^3,4^, particularly for cardiovascular death. Notably, cancer and non-vascular death have accounted for the larger proportion of total deaths in DM patients in recent years^3–5^.

Currently, Sodium-glucose Cotransporter 2 Inhibitor (SGLT2i), which reduce blood sugar levels, are highly effective in preventing diabetic kidney disease progression and cardiovascular diseases in type 2 DM (T2DM) patients. Predominantly due to cardiovascular mechanisms beyond glycemic control, SGLT2i-use facilitates significant risk reductions^6–8^ in all-cause death and cardiovascular death for patients already receiving secondary preventative medication for heart disease in large randomized clinical trials (RCTs). Survival is the primary standard end-point when investigating SGLT2i effects, however, deaths may be too low to show statistically significant effects or identify specific causes of death other than cardiovascular death in these trials. The Dapagliflozin and Prevention of Adverse Outcomes in Chronic Kidney Disease trial^9^ reported that dapagliflozin was associated with reduced non-cardiovascular death, including cancer death. Thus, large database analyses should be encouraged.

In this study, we used Taiwan’s National Health Insurance (NHI) database to determine associations between SGLT2i-use and deaths when compared with Dipeptidyl peptidase 4 inhibitor (DPP4i)-use for T2DM. We further identified the causes of death using the linked National Register of Deaths. Furthermore, we investigated which patient groups could benefit from SGLT2i-use in terms of lowering death risk.

## Methods

### Database information

We used data from the Taiwan National Health Insurance Research Database (NHIRD). In 1995, comprehensive coverage of medical information from approximately 23 million citizens in Taiwan was established by the NHI program. All records, including outpatient care, hospital care, laboratory tests, drug prescriptions, and interventional procedures were collected for medical expense declarations. Also, Taiwan's National Health Research Institutes provided access to these databases for research purposes. Furthermore, databases were linked to the National Death Registry, where causes of death were coded according to the International Classification of Diseases, 10th Revision, Clinical Modification (ICD-10-CM). This research was approved by the China Medical University Hospital ethics committee and performed in agreement with World Medical Association Declaration of Helsinki guidelines.

### Study design and participants

**Figure 1:**
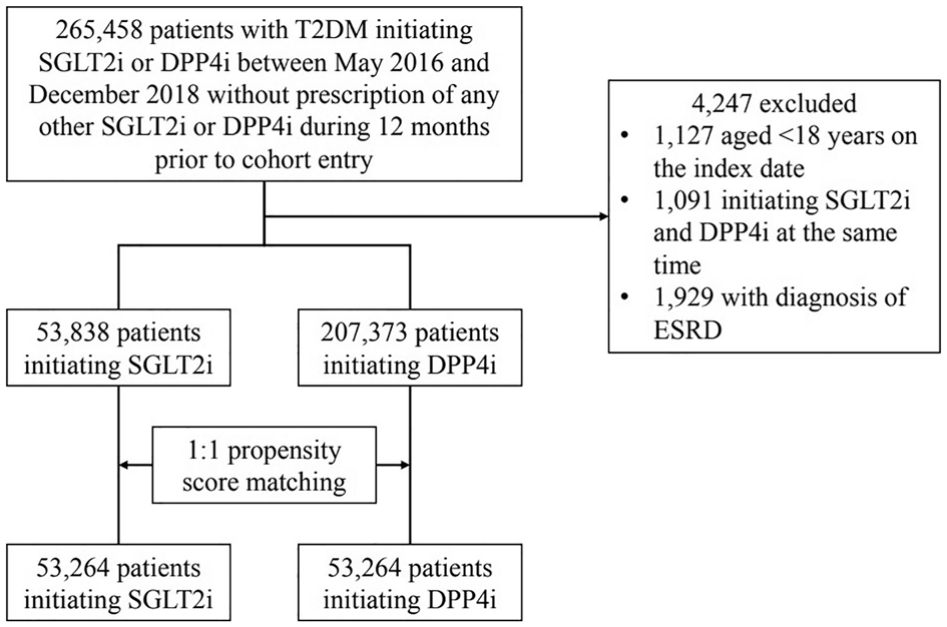
Flow diagram showing patient selection.

We established a nationwide retrospective cohort study and used deidentified secondary data from the NHIRD. All incident patients with T2DM were defined using diagnosis codes from the International Statistical Classification of Diseases, 9th and 10th Revision, Clinical Modification (ICD-9-CM and ICD-10-CM) (Supplementary Table 1). T2DM had to diagnosed at least three times in outpatient clinics or once during hospitalization, within one year. Importantly, diagnostic accuracy was previously verified using this database^10^. We identified 265,458 patients with T2DM who were initially administered SGLT2i or DPP4i between May 2016 (SGLT2i release date in Taiwan) and December 2018. The index study date was defined as the first day of SGLT2i- or DPP4i-use. After excluding individuals < 18 years old and those simultaneously receiving SGLT2i and DPP4i before study entry and undergoing end stage renal disease (ESRD), 53,838 and 207,373 patients were identified as filling SGLT2i or DPP4i prescriptions, respectively, and enrolled. ESRD patients were excluded because SGLT2i is contraindicated in these patients. Furthermore, to avoid the confounding effects of baseline comorbidities and medicine-use, we performed a 1:1 propensity score matching on 53,264 pairs receiving SGLT2i or DPP4i in T2DM patients, based on age, gender, index year, comorbidities, and other medicines. A detailed study flow chart is shown (Fig. 1).

### SGLT2i or DPP4i administration

SGLT2i medicines included dapagliflozin, empagliflozin, and canagliflozin. DPP4i medicines included alogliptin, linagliptin, sitagliptin, saxagliptin, and vildagliptin alone, or metformin combined pills. We collected detailed information on drug type, quantity, dose, dispensing date, and days of drug supply for both SGLT2i and/or DPP4i.

### Definition of outcomes and other covariates

The incidence of overall cause of death in the propensity score matched population was a primary outcome. Deaths were verified by the National Register of Deaths. Further causes of death were determined from death certificates according to ICD-10-CM codes. Causes of death were grouped based on definitions from the U.S. National Center for Health Statistics^3^. All underlying causes of death were grouped into four general categories: all-cause death, cardiovascular death, cancer death, and non-cancer, non-vascular death. In addition, we also assessed death rates for the leading causes of death, including heart diseases, cerebrovascular disease, hypertension and hypertensive renal disease, DM, chronic lower respiratory diseases, nephritis, nephrotic syndrome and nephrosis, chronic liver disease, septicemia, Alzheimer's disease and Parkinson's disease, accidents, and intentional self‐harm. Specific codes for causes of death are shown (Supplementary Table 2).

We compared the distribution of baseline comorbidities, and medicine prescription history between groups receiving SGLT2i or DPP4i. Comorbidities included hypertension, hyperlipidemia, cerebral vascular disease, coronary artery disease, chronic kidney disease, cancer, chronic lower respiratory diseases, chronic liver disease, Alzheimer's disease and Parkinson's disease, and mental disorders which had occurred in at least three ambulatory care visits or one inpatient care visit within one year of the index date. Except for SGLT2i or DPP4i-use, other medicines such as Glucagon-like peptide-1 (GLP-1) agonists, insulin, metformin, aspirin, angiotensin converting enzyme inhibitor/angiotensin II receptor blockers (ACEI/ARB), and statins used before the index date were adjusted in our models. All T2DM patients were followed-up until death or study end, December 31st 2018, or whichever occurred first.

### Statistical analysis

Patient descriptive data were presented according to SGLT2i or DPP4i groups, and included the mean and standard deviation for continuous as well as number and frequency of categorical variables. Standardized mean differences (SMD) were calculated to compare mean differences between two groups. For propensity score matching, propensity scores were determined by multiple logistic regression analysis after adjusting for baseline covariates. SMDs with a cut-off of 0.10 were used to assess the balance of covariates between two groups in the propensity score matched population. We used Kaplan–Meier curves to plot survival curves for SGLT2i and DPP4i users. In addition, we evaluated hazard ratios (HRs) and 95% confidence intervals (CIs) of all-cause death, cardiovascular death, cancer death, and non-cancer, non-vascular death using univariate and multiple Cox proportional hazard regression models. We also preformed stratification analyses by SGLT2i type, including, dapagliflozin, empagliflozin, and canagliflozin. Finally, we performed post hoc subgroup analyses using baseline comorbidities and other medication-use using multiple Cox proportional hazard regression models. Multiplicative interactions between SGLT2i-use and baseline comorbidities and other medications on all-cause death were calculated by adding cross-product terms in multiple Cox proportional hazard regression models. All other statistical analyses were conducted using SAS statistical software (SAS Institute, Cary, NC, USA). Statistical significance was defined as a two-sided *p* < 0.05 value.

### Ethics approval and consent to participate

This study was approved by the Research Ethics Committee of China Medical University Hospital (CRREC-109-018). All methods were performed in accordance with World Medical Association Declaration of Helsinki guidelines.

## Results

### Comorbidity and medicine-use characteristics between SGLT2i and DPP4i users

In the overall population, we identified 53,838 and 207,373 patients with T2DM who had received SGLTi and DPP4i prescriptions. SGLT2i users were younger with less female numbers when compared with DPP4i users (Table 1). Furthermore, T2DM patients receiving SGLT2i displayed higher hyperlipidemia, coronary artery disease, and were taking more GLP-1 agonists, insulin, metformin, statins, aspirin, and ACEI/ARB medications.

**Table 1:** Clinical information of T2DM patients taking DPP4i and SGLT2i in the full cohort and the matched cohort.

	All population			Propensity score matching		
	DPP4i	SGLT2i	SMD	DPP4i	SGLT2i	SMD
	N = 207,373	N = 53,838		N = 53,264	N = 53,264	
Age, Mean ± Std	62.77 ± 12.88	57.75 ± 12.27	− 0.40	57.76 ± 12.43	57.87 ± 12.22	0.01
Gender (Women), N (%)	99,445 (47.95%)	23,626 (43.88%)	0.08	23,614 (44.33%)	23,396 (43.92%)	0.01
**Comorbidity, N (%)**						
Hypertension	24,627 (11.88%)	5076 (9.43%)	0.08	4198 (7.88%)	5013 (9.41%)	− 0.05
Hyperlipidemia	136,356 (65.75%)	38,378 (71.28%)	− 0.12	38,653 (72.57%)	37,951 (71.25%)	0.03
Cerebral vascular disease	4407 (2.13%)	545 (1.01%)	0.09	378 (0.71%)	544 (1.02%)	− 0.03
Coronary artery disease	34,712 (16.74%)	10,823 (20.10%)	− 0.09	10,034 (18.84%)	10,651 (20.00%)	− 0.03
Chronic kidney disease	27,670 (13.34%)	6101 (11.33%)	0.06	5697 (10.70%)	6019 (11.30%)	− 0.02
Cancer	2529 (1.22%)	347 (0.64%)	− 0.06	257 (0.48%)	346 (0.65%)	0.02
Chronic lower respiratory diseases	24,323 (11.73%)	5505 (10.23%)	− 0.05	5075 (9.53%)	5442 (10.22%)	0.02
Chronic liver disease	20,369 (9.82%)	5304 (9.85%)	0.00	5384 (10.11%)	5255 (9.87%)	− 0.01
Alzheimer's Disease and Parkinson's disease	4546 (2.19%)	462 (0.86%)	− 0.11	370 (0.69%)	461 (0.87%)	0.02
Mental disorders	41,375 (19.95%)	8797 (16.34%)	− 0.09	8432 (15.83%)	8712 (16.36%)	0.01
**Diabetes medications, N (%)**						
GLP-1 agonist	400 (0.19%)	908 (1.69%)	− 0.16	371 (0.70%)	531 (1.00%)	− 0.03
Insulin	22,002 (10.61%)	10,817 (20.09%)	− 0.27	9877 (18.54%)	10,392 (19.51%)	− 0.02
Metformin	143,510 (69.20%)	48,260 (89.64%)	− 0.52	48,052 (90.21%)	47,691 (89.54%)	0.02
**Other medications, N (%)**						
Statin	109,579 (52.84%)	33,274 (61.80%)	− 0.18	33,155 (62.25%)	32,844 (61.66%)	0.01
Aspirin	44,784 (21.60%)	13,456 (24.99%)	− 0.08	12,954 (24.32%)	13,251 (24.88%)	− 0.01
ACEI/ARB	103,341 (49.83%)	29,374 (54.56%)	− 0.09	28,958 (54.37%)	28,995 (54.44%)	0.00

To avoid confounding issues with baseline comorbidities and medicine-use, we identified 53,264 matched pairs of SGLT2i and DPP4i users in the propensity score matched population. The mean (SD) duration from T2DM diagnosis to index date was 7.83 (5.60) years in DPP4i and 8.71 (5.64) years in SGLT2i in these population. We observed a similar distribution of age, gender, baseline comorbidities, and medication-use in both groups, as indicated by an SMD cut-off value of 0.10.

### Associations between SGLT2i-use and death risk

**Figure 2:**
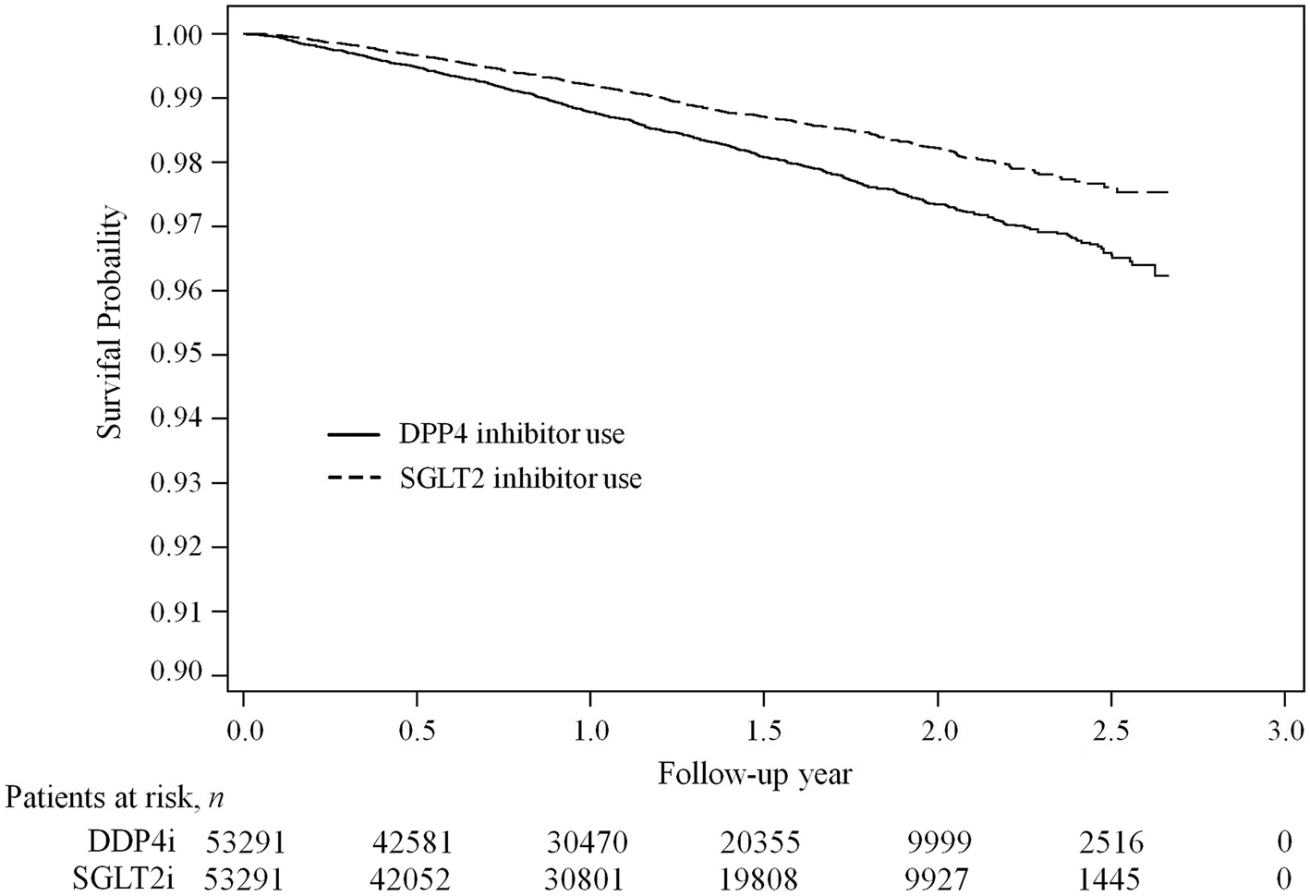
Survival curves for overall mortality in type 2 diabetes mellitus patients using DPP4i and SGLT2i in the propensity score matched population.

Survival analyses during the follow-up period indicated significant differences in the all-cause death rate between SGLT2i and DPP4i users in the propensity score matched population (Fig. 2, Log-rank test p value < 0.01). The all-cause death rate in SGLT2i and DPP4i users was 8.67 and 12.41 per 1000 person-years, respectively.

From univariate Cox regression models, we observed a significant 0.70-fold (95% CI 0.63–0.78), 0.72-fold (95% CI 0.58–0.89), 0.78-fold (95% CI 0.63 –0.96), and 0.66-fold (95% CI 0.57–0.77) of all-cause death, cardiovascular death, cancer death, and non-cancer, non-vascular death, respectively, in patients receiving SGLT2i compared to DPP4i (Table 2). When further adjusted by age, gender, hypertension, hyperlipidemia, cerebral vascular disease, coronary artery disease, chronic kidney disease, diabetes medications usage (GLP-1 agonist, insulin, and metformin), and other medication usages (aspirin, ACEI/ARB and statin), SGLT2i users were similarly associated with a lower risk of all-cause death, cardiovascular death, cancer death, and non-cancer, non-vascular death (HR = 0.66 for all-cause death, HR = 0.68 for cardiovascular death, HR = 0.73 for cancer death, and HR = 0.62 for non-cancer, non-vascular death). In addition, we analyzed associations between different SGLT2i medications (dapagliflozin, empagliflozin, and canagliflozin) and the risk of various death causes. Approximately 55% and 43% of SGLT2i users were receiving dapagliflozin or empagliflozin, respectively. Our data showed that SGLT2i users taking dapagliflozin or empagliflozin had significantly lower associations with the risk of all-cause death, cardiovascular death, cancer death, and non-cancer, non-vascular death (all *p* values < 0.05 from multiple Cox regression models).

**Table 2:** Different causes of mortality risks between patients with DPP4i and with SGLT2i use in the propensity score matched population.

	Events, n	Person-years	Mortality rate per 100 person-years	Crude HR (95% CI)	*p* value	Adjusted HR (95% CI)	*p* value
**Deaths from all causes**							
DPP4i (N = 53,264)	820	66,101.22	12.41	REF		REF	
SGLT2i (N = 53,264)	563	64,908.64	8.67	0.70 (0.63–0.78)	< 0.001	0.66 (0.59–0.74)	< 0.001
Dapagliflozin (N = 29,834)	284	36,357.31	7.81	0.63 (0.55–0.72)	< 0.001	0.65 (0.57–0.75)	< 0.001
Empagliflozin (N = 23,403)	278	28,374.46	9.8	0.80 (0.69–0.91)	< 0.001	0.67 (0.58–0.76)	< 0.001
Canagliflozin (N = 601)	1	176.87	5.65	0.62 (0.09–4.42)	0.63	0.62 (0.09–4.41)	0.63
**Cardiovascular deaths**							
DPP4i (N = 53,264)	205	66,101.22	3.1	REF		REF	
SGLT2i (N = 53,264)	144	64,908.64	2.22	0.72 (0.58–0.89)	0.003	0.68 (0.55–0.84)	< 0.001
Dapagliflozin (N = 29,533)	68	36,357.31	1.87	0.61 (0.46–0.80)	< 0.001	0.63 (0.48–0.84)	0.001
Empagliflozin (N = 23,136)	76	28,374.46	2.68	0.87 (0.67–1.13)	0.30	0.73 (0.56–0.95)	0.02
Canagliflozin (N = 595)	0	176.87	0	–		–	
**Cancer deaths**							
DPP4i (N = 53,264)	199	66,101.22	3.01	REF		REF	
SGLT2i (N = 53,264)	151	64,908.64	2.33	0.78 (0.63–0.96)	0.02	0.73 (0.59–0.90)	0.003
Dapagliflozin (N = 29,533)	81	36,357.31	2.23	0.75 (0.58–0.97)	0.03	0.76 (0.59–0.99)	0.04
Empagliflozin (N = 23,136)	69	28,374.46	2.43	0.82 (0.62–1.07)	0.15	0.68 (0.52–0.90)	0.007
Canagliflozin (N = 595)	1	176.87	5.65	3.18 (0.44–22.76)	0.25	3.24 (0.45–23.38)	0.24
**Non-cancer, non-vascular deaths**							
DPP4i (N = 53,264)	416	66,101.22	6.29	REF		REF	
SGLT2i (N = 53,264)	268	64,908.64	4.13	0.66 (0.57–0.77)	< 0.001	0.62 (0.53–0.72)	< 0.001
Dapagliflozin (N = 29,533)	135	36,357.31	3.71	0.59 (0.49–0.72)	< 0.001	0.61 (0.50–0.74)	< 0.001
Empagliflozin (N = 23,136)	133	28,374.46	4.69	0.75 (0.62–0.91)	0.004	0.63 (0.52–0.76)	< 0.001
Canagliflozin (N = 595)	0	176.87	0	–		–	

Adjusted models were adjusted by age, gender, hypertension, hyperlipidemia, cerebral vascular disease, coronary artery disease, chronic kidney disease, diabetes medications usage (GLP-1 agonist, insulin, and metformin), and other medication usages (aspirin, ACEI/ARB and statin).

**Figure 3:**
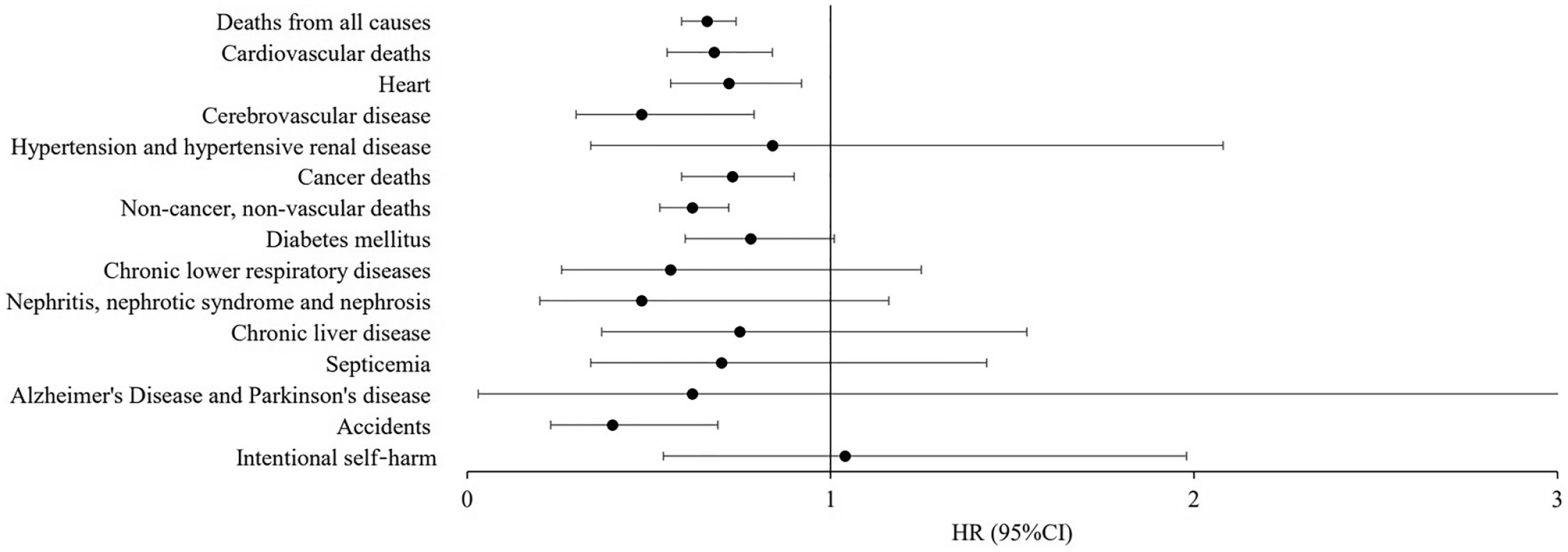
Death risks in type 2 diabetes mellitus patients prescribed SGLT2i versus DPP4i, according to specific causes of death.

We also performed an association analysis for specific causes of death risk (Fig. 3). We observed a significantly lower death risk for heart diseases (HR = 0.72; 95% CI 0.56–0.92), cerebrovascular diseases (HR = 0.48; 95% CI 0.30–0.79), and accidents (HR = 0.40; 95% CI 0.23–0.69) with SGLT2i in the propensity score matched population.

Finally, post hoc subgroup analyses and interaction analyses of various comorbidities and other medicines and SGLT2i-use on death risk were performed (Supplementary Fig. 1). SGLT2i users were mostly associated with a lower risk of all-cause death in post hoc subgroup analyses for age, sex, baseline comorbidities, and medication use. Among characteristics, statistical interactions were identified between SGLT2i-use and patients receiving ACEI/ARB for all-cause death risk (interaction *p* = 0.02). This suggested that associations between SGLT2i-use with overall death risk were affected by ACEI/ARB-use. SGLT2i was associated with lower death risk in non-users of ACEI/ARB -based treatments at baseline than ACEI/ARB users. Other comorbidities and medicine-use did not statistically interact with SGLT2i-use for all-cause death risk (all *p* > 0.05). Thus, SGLT2i benefits in T2DM patients in terms of lowered death risks were not different across subgroups. We did not perform other subgroup analyses for specific causes of death risk because of the limited number of deaths.

## Discussion

In this study, SGLT2i-use in T2DM was significantly associated with a decreased risk of death when compared with DPP4i-use in a national cohort in Taiwan. After adjusting for potential confounders using propensity score matching, a significantly lower risk for all-cause death of 34% with SGLT2i-use was identified. In addition to cardiovascular death, SGLT2i-use was also associated with a lower risk for cancer death and non-cancer, non-vascular death. The benefits of SGLT2i-use for a lower death risk were not different across subgroups.

Our study indicated an association between SGLT2i-use and a lower risk of all-cause death in a real-world database, in agreement with data from RCTs^6–8^. Other population-based studies^11,12^ also showed a reduced risk of all-cause death for SGLT2i therapy when compared with other glucose-lowering drugs. Here, we chose DPP4i as a comparator because all DPP4i-types displayed neutral effects toward all-cause death or cardiovascular death^13^. Survival is the most important clinical endpoint when conducting a new medicine trial^14^. Similarly, death prevention is one of the most important public health outcomes of any treatment or preventative intervention type. Therefore, the association of SGLT2i-use with decreased risk of death should be emphasized in clinical settings.

Several factors likely accounted for the low death incidence rate during SGLT2i-use. SGLT2i-use was previously shown to exert beneficial effects toward cardiovascular death^13^. From a previous meta-analysis, SGLT2i appeared to mitigate 15% of cardiovascular death and 11% of myocardial infarctions^13^. In our study, we observed a 32% lower risk of cardiovascular death with SGLT2i-use, including a 28% lower risk of death from heart disease and 52% lower risk from cerebrovascular disease. SGLT2i heart benefits may be due to multiple mechanisms beyond glycemic control^15^. A recent study reported that SGLT2i was associated with a significant reduction in hemorrhagic stroke^16^. Furthermore, an animal study indicated that SGLT2i generated protective effects after acute ischemic stroke by suppressing inflammation and oxidative stress^17^.

We observed a 0.73-fold decreased risk of cancer death in patients receiving SGLT2i. In a previous study, the relative risk reduction for cancer death was 58% with dapagliflozin^9^. Diabetes is associated with an increased risk of cancer incidence and death^18^, and cancer deaths become as the leading cause of diabetes-related deaths in recent years^4^. In a previous RCT, SGLT2i was not associated with risk of all-cancer incidence^19^. However, a recent study indicated a benefit of SGLT2i in suppressing cell growth^20^. SGLT2i was also added to standard cancer therapies in human studies^21,22^, and currently, clinical trials investigating SGLT2i safety and efficacy in cancer treatment are underway^20^. These informative studies suggest SGLT2i antitumor abilities should be thoroughly investigated in further comprehensive studies.

Our study is the first to indicate associations between SGLT2i-use and a lower risk of non-cancer, non-vascular death. It was surprising that SGLT2i-use was associated with decreased death from accidents. In previous work, SGLT2i improved life quality, including pain, mental health, and general health perceptions^23^. Because poor functional status was considered a predictor of future-fall risk^24^, SGLT2i possibly reduced accidents by improving life quality and functional status. We observed the tendency that SGLT2i-use benefits for a lower death risk from multiple diseases, despite insignificance. Increasing evidence has indicated that SGLT2i exerts protective effects on nephritis, nephrotic syndrome and nephrosis^25^, chronic liver disease ^26^, and septicemia survival^27^. SGLT2i-use also demonstrated consistent heart and kidney protective effects in patients with obstructive sleep apnea^28^ and chronic obstructive pulmonary disease^29^.

Subgroup analyses of baseline characteristics suggested SGLT2i protective effects were not different across subgroups. Our findings agreed with previous studies showing that dapagliflozin reduced the risk of death across a broad spectrum of age^30^, heart failure^31^, and metformin-use^32^. The reduction of all-cause death was considered a class effect between different SGLT2i types in previous research^33^, and we demonstrated consistent protective effects of empagliflozin and dapagliflozin toward cancer death and non-cancer, non-vascular death. One exception was that this study indicated protection effect of SGLT2i was less in ACEI/ARB users than non-users (interaction *p* < 0.05). Fortunately, a beneficial association between SGLT2i-use and a lower risk of death was significant in ACEI/ARB users.

Our study data were strengthened by the large sample size and nationwide scope covering > 99% of 23 million Taiwanese citizens. By using a national cohort study linked to the National Register of Deaths, we precisely studied death in SGLT2i-users, and causes of death based on ICD-10-CM codes. Furthermore, we grouped specific causes of death based on clear definitions from the National Center for Health Statistics^3^. However, our study was also limited in some respects. Firstly, the finding that fewer deaths due to cancer had occurred in SGLT2i-users should be cautiously interpreted; our study was not primarily designed to survey cancer deaths, and many potential confounding factors were not considered. Secondly, a cause of death report is formulated by medical opinion and subjective judgment; thus, potential misclassifications cannot be ruled out. Thirdly, SGLT2i-use was approved in 2016 in Taiwan; therefore, the outcome observation period was short. In addition, the decreased sample size and event rate were in the analysis of specific cause of death, which would not have had much power to detect differences. Next, it is unclear what the accident specifically indicates and its association with SGLT2i needed further study to explore it. Lastly, detailed laboratory values (e.g. HbA1C levels) were not included in the NHI database, potentially influencing study outcomes. Further studies with longer follow-up times and higher patient numbers are warranted.

## Conclusions

In T2DM patients, we identified an association between SGLT2i and a lower risk of death when compared with DPP4i-use. The potential beneficial effects of SGLT2i on cancer death and non-cancer, non-vascular death must be comprehensively investigated in future studies.

## Supplementary Information

Supplementary Information 1.

Supplementary Information 2.

## Data Availability

The data that support the findings of this study are available from the Taiwan National Health Insurance Research Database (NHIRD) but restrictions apply to the availability of these data, which were used under license for the current study, and so are not publicly available. Any detail for data requests can be through the NHIRD (http://nhird.nhri.org.tw).

## Abbreviations

SGLT2i: Sodium-glucose co-transporter 2 inhibitors
T2DM: Type 2 diabetes mellitus
DPP4i: Dipeptidyl peptidase 4 inhibitors
NHRI: National Health Research Institute
GLP-1: Glucagon-like peptide-1
NHIRD: National Health Insurance Research Database
