# Supplementary material for: Association of SGLT2 inhibitors with lower incidence of death in type 2 diabetes mellitus and causes of death analysis
Source: Sci Rep. 2022 Jun 16;12:10147. doi: 10.1038/s41598-022-13760-7 (PMC9203810; doi:10.1038/s41598-022-13760-7)
Supplement: Supplementary file 1 — Supplementary Information 1. [file 41598_2022_13760_MOESM1_ESM.docx]

Supplementary Table 1. Covariates used for cohort creation

| Type 2 Diabetes Mellitus | ICD-09: "250.0","250.00","250.02","250.10","250.12","250.2","250.20","250.22","250.30","250.32","250.40","250.42","250.50","250.52","250.60","250.62","250.70","250.72","250.80","250.82","250.90","250.92".  ICD-10:  "E11","E110","E110.0","E110.1","E112","E112.1","E112.2","E112.9","E113","E113.1","E113.11","E113.19","E113.2","E113.21","E113.29","E113.3","E113.31","E113.39","E113.4","E113.41","E113.49","E113.5","E113.51","E113.59","E113.6","E113.9","E114","E114.0","E114.1","E114.2","E114.3","E114.4","E114.9","E115","E115.1","E115.2","E115.9","E116","E116.1","E116.10","E116.18","E116.2","E116.20","E116.21","E116.22","E116.28","E116.3","E116.30","E116.38","E116.4","E116.41","E116.49","E116.5","E118","E119". |
| --- | --- |

Supplementary Table 2. ICD-10 codes for specific causes of death

| Causes of death | ICD-10 codes |
| --- | --- |
| All-cause deaths | A00-Y98 |
| Cardiovascular deaths | I00-I99 |
| Heart | I00‐I09, I11, I13, I20‐I51 |
| Cerebrovascular disease | I60-I69 |
| Hypertension and hypertensive renal disease | I10, I12 |
| Cancer deaths | C00‐C97 |
| Non-cancer, non-vascular deaths | (A00-Y98) - (I00-I99) - (C00‐C97) |
| Diabetes mellitus | E10‐E14 |
| Chronic lower respiratory diseases | J40‐J47 |
| Nephritis, nephrotic syndrome and nephrosis | N00‐N07, N17‐N19, N25‐N27 |
| Chronic liver disease | K70, K73‐K74 |
| Septicemia | A40‐A41 |
| Alzheimer's Disease and Parkinson's disease | G20‐G21, G30 |
| Accidents | V01‐X59, Y85‐Y86 |
| Intentional self‐harm | X60–X84, Y87.0 |
