# Supplementary figures and images for: Association of SGLT2 inhibitors with lower incidence of death in type 2 diabetes mellitus and causes of death analysis
Source: Sci Rep. 2022 Jun 16;12:10147. doi: 10.1038/s41598-022-13760-7 (PMC9203810; doi:10.1038/s41598-022-13760-7)

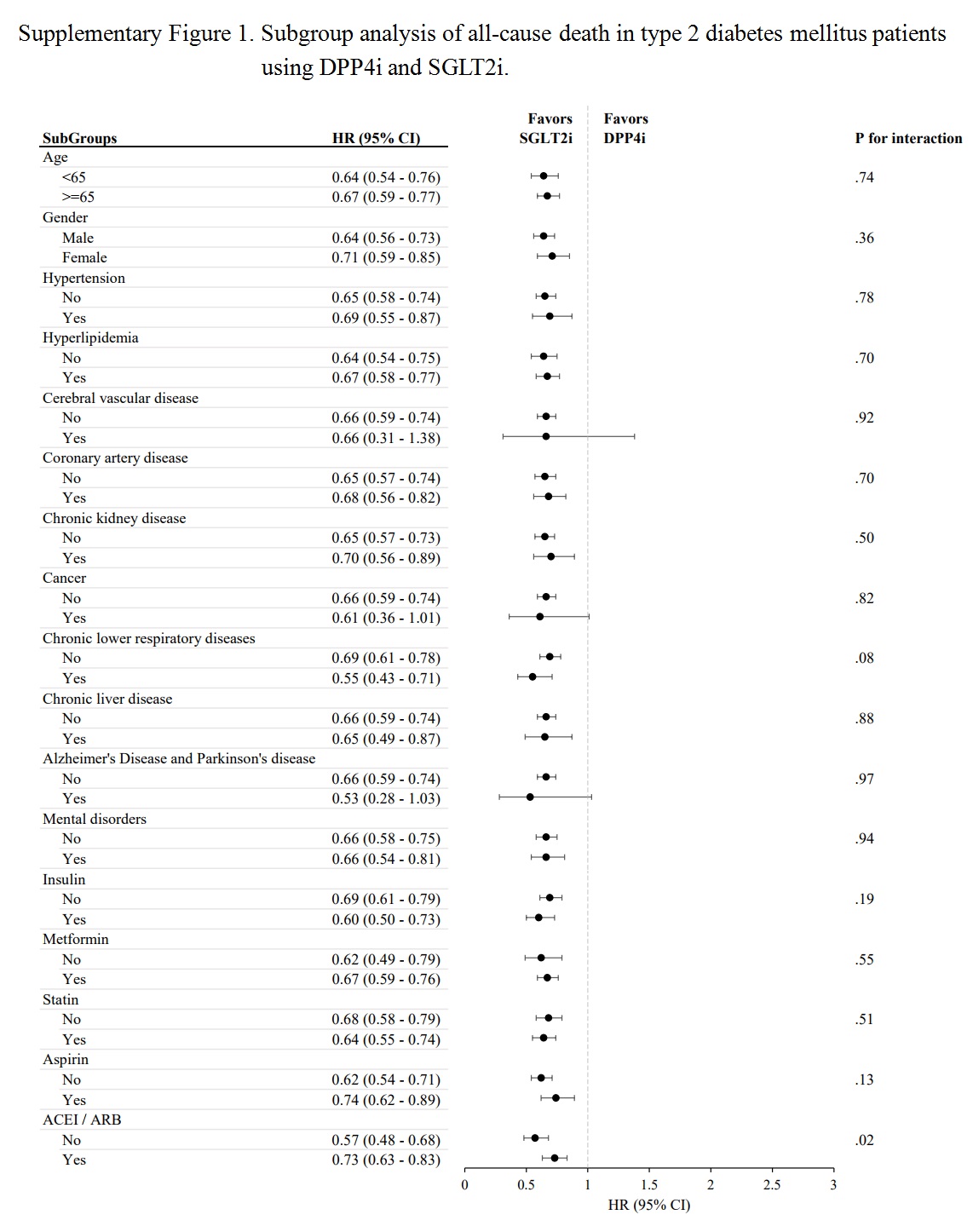

Supplement: Supplementary file 2 — Supplementary Information 2. [file 41598_2022_13760_MOESM2_ESM.jpg]
